# Atomistic Construction of Silicon Nitride Ceramic Fiber Molecular Model and Investigation of Its Mechanical Properties Based on Molecular Dynamics Simulations

**DOI:** 10.3390/ma16186082

**Published:** 2023-09-05

**Authors:** Yiqiang Hong, Yu Zhu, Youpei Du, Zhe Che, Guoxin Qu, Qiaosheng Li, Tingting Yuan, Wei Yang, Zhen Dai, Weijian Han, Qingsong Ma

**Affiliations:** 1 Science and Technology on Advanced Ceramic Fibers & Composites Laboratory, College of Aerospace Science, National University of Defense Technology, Changsha 410073, China; 2 Beijing System Design Institute of Mechanical-Electrical Engineering, Beijing 100871, Chinayangwei851023@163.com (W.Y.);; 3 The Fourth Academy of CASIC, Beijing 100028, China; 4 Key Laboratory of Science and Technology on High-Tech Polymer Materials, Department of Polymer Chemistry and Physics, Institute of Chemistry, Chinese Academy of Sciences, Beijing 100190, China

**Keywords:** ceramic fiber, amorphous and crystal, silicon nitride, Monte Carlo, tensile fracture property

## Abstract

Molecular simulations are currently receiving significant attention for their ability to offer a microscopic perspective that explains macroscopic phenomena. An essential aspect is the accurate characterization of molecular structural parameters and the development of realistic numerical models. This study investigates the surface morphology and elemental distribution of silicon nitride fibers through TEM and EDS, and SEM and EDS analyses. Utilizing a customized molecular dynamics approach, molecular models of amorphous and multi-interface silicon nitride fibers with complex structures were constructed. Tensile simulations were conducted to explore correlations between performance and molecular structural composition. The results demonstrate successful construction of molecular models with amorphous, amorphous–crystalline interface, and mixed crystalline structures. Mechanical property characterization reveal the following findings: (1) The nonuniform and irregular amorphous structure causes stress concentration and crack formation under applied stress. Increased density enhances material strength but leads to higher crack sensitivity. (2) Incorporating a crystalline reinforcement phase without interfacial crosslinking increases free volume and relative tensile strength, improving toughness and reducing crack susceptibility. (3) Crosslinked interfaces effectively enhance load transfer in transitional regions, strengthening the material’s tensile strength, while increased density simultaneously reduces crack propagation.

## 1. Introduction

Silicon nitride ceramic fibers possess outstanding properties [1], such as high strength, high modulus, low density, high temperature resistance, oxidation resistance, and corrosion resistance [2]. These attributes make them an ideal reinforcement for high-performance ceramic matrix composites and a preferred material for electromagnetic wave penetration in high-temperature environments. As a dual-phase ceramic fiber that combines structural and functional characteristics, silicon nitride ceramic fibers have a wide range of applications in the aerospace industry [3].

Currently, the industrial preparation of silicon nitride fibers can be divided into two approaches based on the differences in precursor materials used. The first approach involves using polysilazane as a precursor [4], and the fiber is prepared through processes such as spinning, non-melting treatment, pyrolysis [5], and high-temperature sintering. This method yields silicon nitride fibers with consistent composition, making it easier to obtain fibers with high strength and low defects [6]. The second approach utilizes polycarbosilane as a precursor and follows a representative process route that includes spinning, non-melting treatment, nitridation and decarburization, and high-temperature sintering. Since there are no carbon atoms in the inherent structure, additional decarburization and nitridation are required to form the silicon nitride structure. Consequently, the resulting silicon nitride fibers from this approach tend to have more defects. Therefore, the different process routes lead to significant differences in the composition, structure, and properties of the silicon nitride fibers [7].

Researchers aiming to comprehensively understand the composition, structure, and properties of silicon nitride ceramic fibers [8], as well as reveal the structure–property relationships to guide material design and optimization, often perform in-depth characterization at multiple levels and scales. This includes analyzing the material’s elemental composition, morphology, crystal and grain boundary structure, as well as the characteristics and distribution of amorphous structures [9]. Among these microstructural features, amorphous and crystalline structures are essential components, making their study a crucial aspect of silicon nitride fiber research. Such investigations hold significant importance in driving the development and innovation of fibers [10].

The crystal structure in fibers represents a periodic region with long-range ordered structure, and the arrangement of atoms in the crystal lattice plays a crucial role in the mechanical properties of materials [11], such as tensile strength [12], compressive strength, and fracture behavior. Moreover, ceramic fibers with high melting points and thermally stable crystal structures tend to maintain their structural and performance stability in high-temperature environments [13]. Additionally, the arrangement of atoms and the energy band structure in the crystal have a significant impact on thermal conductivity [14], insulation, and optical properties. Therefore, the study of crystal structures in fibers is of utmost importance. Researchers have established comprehensive bottom-up characterization methods, such as X-ray diffraction, transmission electron microscopy, XPS, and AFM [15], to investigate crystal structures.

However, characterizing amorphous structures is more challenging than crystalline structures in research, mainly due to the following reasons:

1. Diversity and complexity of structure: Amorphous structures lack regularity and periodicity, leading to high diversity and complexity in their short-range atomic arrangements. As a result, studying amorphous structures often requires a combination of various methods and techniques to describe and characterize their structural features.
2. Nonperiodicity and locality: Unlike crystalline structures, amorphous structures are nonperiodic, making it challenging to use traditional crystallographic methods for analysis and characterization. Additionally, amorphous structures typically exhibit locality, meaning structural features present nonuniform distribution in space, making accurate description and quantification more difficult.
3. Lack of structural descriptors: Due to the diversity and complexity of amorphous structures, it is often challenging to describe them using simple structural models and crystallographic parameters.
4. Experimental technique limitations: Conventional structural characterization techniques such as X-ray diffraction and electron diffraction often have limited capabilities for analyzing amorphous structures. Moreover, amorphous structures often display a wide range of structural scales and irregularities, which exceed the resolution range of traditional techniques.

Traditional experimental techniques may not provide a comprehensive understanding of the intricate atomic interactions and dynamic processes that govern the mechanical response and failure mechanisms of ceramic fibers. As a result, computational approaches like MD simulations have become indispensable tools for studying and elucidating the fundamental aspects of these materials [16]. MD simulations [17,18,19,20] allow researchers to investigate the behavior of ceramic fibers at the atomic scale by tracking the trajectories of individual atoms over time.

This paper starts from experimental characterization results and, based on the composition and distribution of ceramic fiber elements, achieves the construction of amorphous silicon nitride structures and full atomic models of ceramic fibers through a self-assembled molecular model algorithm with spatial atom labeling. It provides an in situ atomic-scale description and simulation of the material’s microstructure and dynamic behavior. Through molecular dynamics simulation, the mechanical behavior of amorphous structures and ceramic fiber structures under external loading, such as stress–strain relationships, was further investigated, providing a detailed atomic-level description and simulation of silicon nitride ceramic fiber’s structural characterization. Molecular simulation, as an advanced research tool, offers an excellent atomic-scale research strategy for studying both amorphous and crystalline structures in the field of ceramics.

## 2. Experimental

### 2.1. Materials and Preparation

Silicon nitride ceramic fibers was purchased from Chengdu Kelong Chemical Co., Chengdu City, China. Either no treatment or a mild surface treatment was performed to avoid any possible effect from changes in the fiber surface and inner structure.

### 2.2. Measurements

Fracture surface of the hybrids was observed on a Hitachi S-4800 scanning electron microscope (SEM, Hitachi, Tokio, Japan) at an accelerating voltage of 10 kV. Transmission electron microscopy (TEM) study was performed using a JEM 2010 TEM microscope (JEOL, Tokyo, Japan).

## 3. Results and Discussion

### 3.1. Structural Characterization and Molecular Dynamics Model Construction of Silicon Nitride Fiber

#### 3.1.1. Construction Algorithm of Amorphous Silicon Nitride Ceramic Fiber Model

Amorphous silicon nitride fibers are noncrystalline materials with no regular periodic arrangement in their molecular structure [21]. They exhibit a certain degree of randomness and disorder. However, the ratio of nitrogen atoms to silicon atoms in the structure can be precisely controlled through chemical synthesis methods, allowing for accurate tuning of their structure and properties. Silicon is one of the main components of amorphous silicon nitride, forming the primary backbone of the fiber structure through the existence of Si-N bonds. The presence of nitrogen not only enhances the diversity and stability of the fiber structure but also regulates the electronic and conductive properties of the fibers. Additionally, depending on the fabrication methods, amorphous silicon nitride structures may also contain carbon (C) and oxygen (O) elements. During the heat treatment process, many amorphous silicon nitride fibers undergo a continuous transformation into α-Si3N4 crystals [22], serving as a reinforcement phase with excellent tensile strength, stiffness, and elastic modulus. When these crystalline structures are in situ generated and combined with the amorphous matrix within the appropriate compositional range, their strength can be transferred to the entire ceramic fiber, enhancing the overall material strength. This imparts the ability to resist stress concentration and crack propagation induced by external loads, thus increasing the tensile and bending strength of the material.

**Figure 1:**
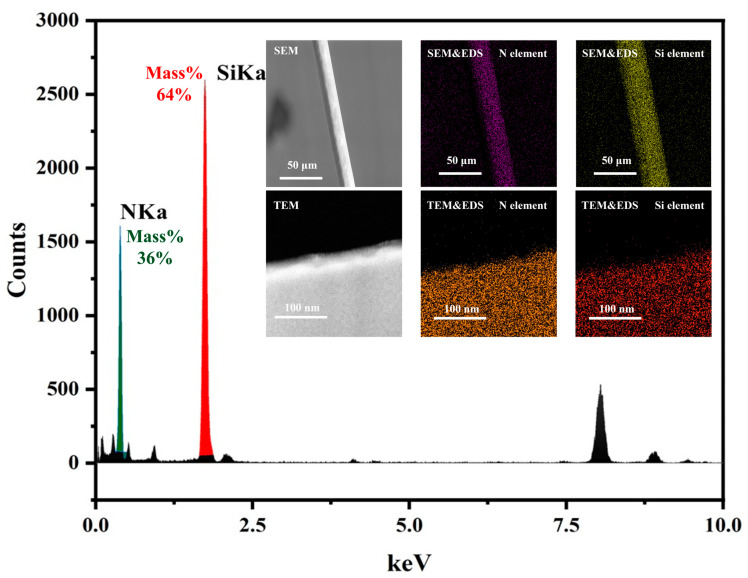
TEM and EDS, and SEM and EDS element content and distribution map of silicon nitride fiber.

As shown in Figure 1, TEM and SEM were used to construct fiber models at different characterization scales, revealing that the samples used in the experiments consist of uniformly distributed Si and N elements. Additionally, no apparent crystal structure was observed in TEM, indicating a uniform distribution of amorphous structure in the samples. Therefore, the molecular models constructed in this study are limited to the following three types:

1. Mixed structure with non-crosslinked interfaces: This model comprises Si and N elements forming short-range ordered and irregular structures based on Si-N bonds, where Si atoms are hybridized in SP3 and N atoms in SP2.
2. Mixed structure with crosslinked interfaces: This model consists of Si and N elements, with some regions existing in the form of α-Si3N4 crystallites surrounded by continuous amorphous Si-N structures. A crosslinking algorithm was applied to bond connectable atoms within 2 Å of any atom in the crystallite, constructing a continuous interface between the two phases.
3. Model with amorphous structure: This model is composed of Si and N elements, with some regions existing in the form of α-Si3N4 crystallites surrounded by continuous amorphous Si-N structures. The amorphous atoms within 2 Å of any atom in the crystallite were removed to ensure phase discontinuity.

**Figure 2:**
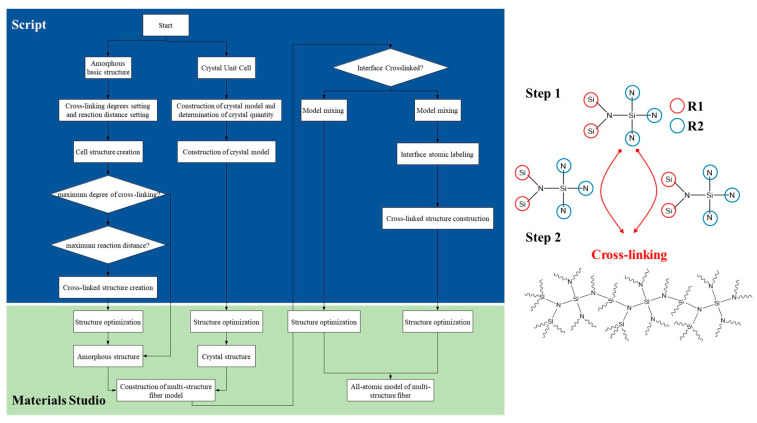
Construction algorithm flowchart and elementary structure of amorphous silicon nitride ceramic fiber model.

A customized crosslinking algorithm [23] was used for the construction of three types of crosslinked structures, with the basic description of the crosslinking algorithm outlined as follows:

Step S1: Construction of molecular models for amorphous and crystalline phases, followed by optimization of the extracted molecular models: the molecular model for the amorphous monomer is constructed, as shown in the Figure 2, where silicon atoms are labeled as R1, and nitrogen atoms are labeled as R2.

Step S2: Creation of N amorphous periodic cells, with selection of the lowest energy configuration. Each cell contains n1 molecular models of the amorphous structure. Structural optimization is performed on all periodic cells using the Forcite module with the COMPASS force field. The van der Waals and electrostatic interactions are handled with the atom-based and Ewald nonbonded summation methods. The smart minimizer method is then employed for structural optimization of the periodic cell system, and the cell with the lowest energy configuration is selected. The initial optimization temperature for the amorphous periodic cells is set to 300 K, with a density of 0.8 g/cm^3^. The specific range of initial temperature (300–800 K) is chosen based on the actual solidification reaction temperature for favorable molecular motion and reasonable model construction. The density is set within the range of 0.5–1.0 g/cm^3^, with an initial density setting of 0.5 g/cm^3^ being more conducive to molecular motion and crosslinking reactions.

Step S3: In the Perl script program, the algorithm is set to achieve dynamic crosslinking based on the actual crosslinking mechanism of the phenylene dinitrile resin, resulting in molecular models with different degrees of crosslinking. The specific steps for dynamic crosslinking are as follows: a. Set the target degree of crosslinking, maximum cutoff radius Rmax, initial cutoff radius Rn, and maximum iteration steps. b. Traverse all A1 and B1 pairs in the cells to determine if the distance Rnow between any A1 and B1 is ≤Rmax and if the iteration steps are ≤the maximum iteration steps. If both conditions are met and there are connectable atomic pairs within the reaction radius with reasonable chemical bonds and topological structures, crosslinking is performed. The crosslinked structure is then optimized and equilibrated through structure optimization and equilibrium simulation. c. Check if the degree of crosslinking is less than or equal to the target degree. If not, repeat step b until the target degree of crosslinking is reached to obtain the desired crosslinked model. Different degrees of crosslinking can be obtained by varying the target degree of crosslinking, maximum cutoff radius Rmax, and maximum iteration steps.

The specific methods and parameters for structural optimization and equilibrium simulation of the cells and crosslinked structures can be adjusted according to the actual needs. In the COMPASS force field, the models are subjected to 500 ps of NVT ensemble and 500 ps of NPT ensemble equilibrium simulations, with a temperature of 800 K and a pressure of atmospheric pressure 1.0 × 10^−4^ GPa. The time step is set to 1 fs to obtain periodic cells. The periodic cells are then subjected to an additional 500 ps of NPT ensemble and 500 ps of NVT ensemble equilibrium simulations at 600 K, with a pressure of atmospheric pressure 1.00 × 10^−4^ GPa.

In this simulation, we conducted canonical ensemble simulations using the number, volume, and temperature (NVT) integration method for a duration of 10 ps. This approach is commonly referred to as constant temperature molecular dynamics (MD). Prior to that, we performed isothermal–isobaric ensemble simulations using the number, pressure, and temperature (NPT) integration method for 100 ps. This allowed us to establish a defined temperature and achieve pressure equilibrium with the environment. In all cases, a time step of 0.001 ps was applied.

To regulate and control the temperature during these simulations, we employed the Nose–Hoover thermostat. The functional forms utilized in our force field are consistent with those found in the COMPASS force field using Equation (1).

(1) $$\begin{array}{ll} & E=\sum_{\mathrm{bond}\;}\left[K_{b2}{\left(b-b_{o}\right)}^{2}+K_{b3}{\left(b-b_{o}\right)}^{3}+K_{b4}{\left(b-b_{o}\right)}^{4}\right]\\ & +\sum_{\mathrm{angle}\;}\left[K_{a2}{\left(\theta -\theta_{o}\right)}^{2}+K_{a3}{\left(\theta -\theta_{o}\right)}^{3}+K_{a4}{\left(\theta -\theta_{o}\right)}^{4}\right]\\ & +\sum_{\mathrm{torsion}\;}\left[K_{t1}(1-cos\phi )+K_{t2}(1-cos2\phi )+K_{t3}(1-cos3\phi )\right]\\ & +\sum_{\mathrm{oopA}\;}K_{\chi }{\left(\chi^{-}\chi_{o}\right)}^{2}\\ & +\sum_{\mathrm{bond}/\mathrm{bond}\;}K_{bb}\left(b-b_{o}\right)\left(b^{\prime }-b_{o}^{\prime }\right)\\ & +\sum_{\mathrm{bond}/\mathrm{angle}\;}K_{ba}\left(b-b_{o}\right)\left(\theta -\theta_{o}\right)\\ & +\sum_{\mathrm{angle}-\mathrm{angle}\;}K_{aa}\left(\theta -\theta_{o}\right)\left(\theta^{\prime }-\theta_{o}^{\prime }\right)\\ & +\sum_{\mathrm{bond}/\mathrm{torsion}\;}\left(b-b_{o}\right)\left[K_{bt1}cos\phi +K_{bt2}cos2\phi +K_{bt3}cos3\phi \right]\\ & +\sum_{\mathrm{angle}/\mathrm{torsion}\;}\left(\theta -\theta_{o}\right)\left[K_{at1}cos\phi +K_{at2}cos2\phi +K_{at3}cos3\phi \right]\\ & +\sum_{\mathrm{angle}/\mathrm{torsion}\;/\;\mathrm{angle}\;}k\left(\theta -\theta_{o}\right)\left(\theta^{\prime }-\theta_{o}^{\prime }\right)\left(\phi -\phi_{o}\right)\\ & +\sum_{\mathrm{nonbond}\;}\left\{\varepsilon_{ij}\left[{\left(\frac{r_{ij}^{o}}{r_{ii}}\right)}^{9}-3{\left(\frac{r_{ij}^{o}}{r_{ii}}\right)}^{6}\right]+\frac{q_{i}q_{j}}{4\pi \varepsilon_{n}r_{ii}}\right\} \end{array}$$

The structures were subjected to strain along three orthogonal Cartesian coordinates: direction-1, direction-2, and direction-3. This was carried out at a temperature of 298 K and strain rates of 1010 s^−1^ using a time step of 0.001 ps. The applied stresses on the structures were calculated using Equation (2), which is derived from the virial stress theorem.

(2) $$\sigma_{ij}^{\alpha }=\frac{1}{\Omega^{\alpha }}\left(\frac{1}{2}m^{\alpha }\nu_{i}^{\alpha }\nu_{j}^{\alpha }+\sum_{\beta =1}^{n}r_{\alpha \beta }^{i}f_{\alpha \beta }^{j}\right)$$

where i,j=Cartesian coordinate system indices;

α,β $=Atomic\;indices\;m^{\alpha }=Mass\;of\;the\;atoms$;
$v^{\alpha }=$ Velocity of the atoms;
rαβ= Atomic distance between α and β;
fαβ= Atomic force between α and $\beta \Omega^{\alpha }=$ Volume of atom α.

#### 3.1.2. Construction Algorithm of Molecular Model Construction of Amorphous and Crystal Structure Transition Interface

**Figure 3:**
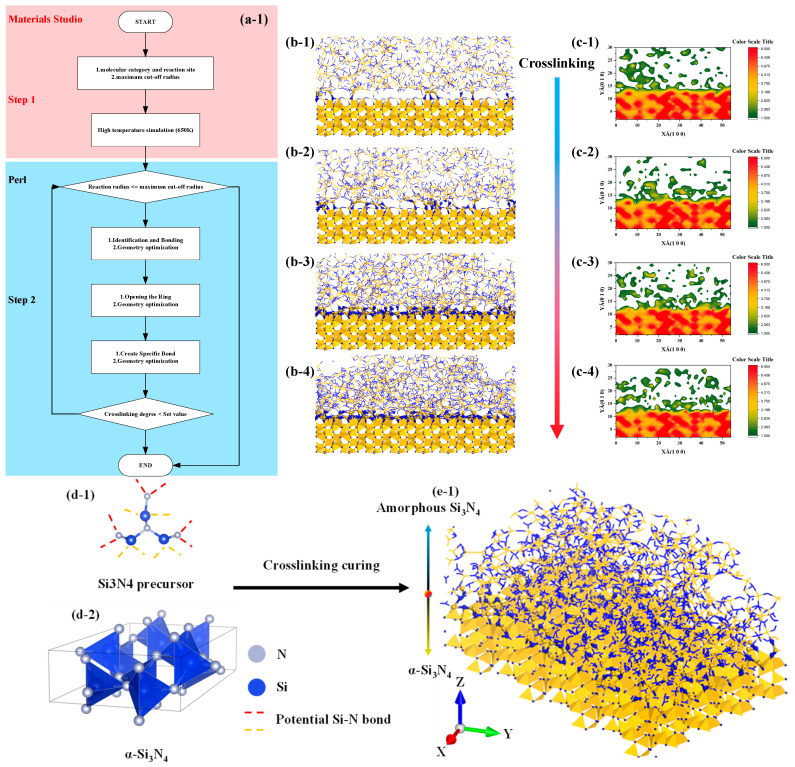
Flow chart and structure diagram of molecular model construction of amorphous and crystal structure transition interface, the color standard is based on the actual image. (**a1**): Flow chart of structure generation algorithm, (**b1**–**b4**): interface structure generation diagram, (**c1**–**c4**): interface structure density projection diagram, (**d1**,**d2**): raw material structure diagram, (**e1**): interface structure model diagram.

As shown in the Figure 3, the algorithm for describing the transition structure between amorphous and crystalline interfaces can be outlined as follows:

1. Determine the models for the crystal and amorphous materials: Select appropriate structures for both the crystal and amorphous materials. In this study, the crystal structure chosen is α-Si3N4.
2. Identify the interface region: Define the contact interface region between the crystal and amorphous materials, as this is the main focus of the transition structure. In this study, the spatial region within 20 Å above the interface is considered the primary bonding region, while the preexisting crystal structure is set to be nonbonding.
3. Generate the interface atom structure: Within the interface region, utilize the previously described crosslinking procedure to construct a crosslinking reaction model. Apply a criterion for bond formation and cancel bonding when the bonding atoms are beyond 20 Å from the interface and vice versa.
4. Energy minimization and structure optimization: Perform energy minimization and structure optimization on the generated interface atom structure to achieve a relatively stable state. This step helps eliminate any unreasonable structures and brings the interface atom structure closer to actual physical phenomena.

#### 3.1.3. Construction Algorithm of Molecular Model Construction of Amorphous and Crystal Structure Transition Interface

**Figure 4:**
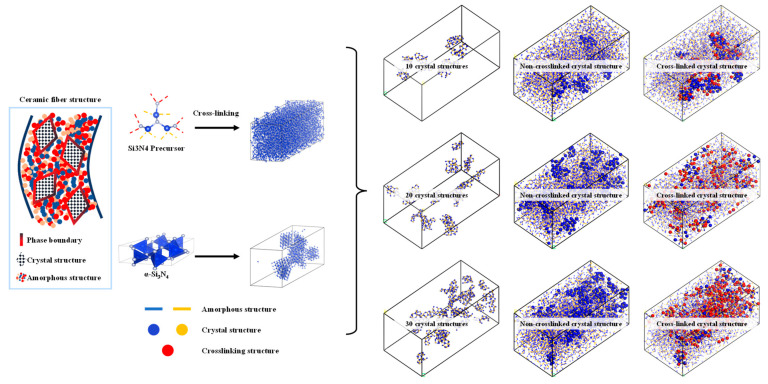
Construction of structure diagram using mixed molecular model of amorphous and crystal structure.

As shown in Figure 4, the algorithm for describing the transition structure between amorphous and crystalline interfaces can be outlined as follows:

1. Determine the models for the crystal and amorphous materials: Select appropriate structures for both the crystal and amorphous materials. In this study, the crystal structure chosen is α-Si3N4.
2. Identify the interface region: Define the contact interface region between the crystal and amorphous materials, as this is the main focus of the transition structure. In this study, the spatial region within 20 Å above the interface is considered the primary bonding region, while the preexisting crystal structure is set to be nonbonding.
3. Generate the interface atom structure: Within the interface region, utilize the previously described crosslinking procedure to construct a crosslinking reaction model. Apply a criterion for bond formation and cancel bonding when the bonding atoms are beyond 20 Å from the interface and vice versa.
4. Energy minimization and structure optimization: Perform energy minimization and structure optimization on the generated interface atom structure to achieve a relatively stable state. This step helps eliminate any unreasonable structures and brings the interface atom structure closer to actual physical phenomena.

The molecular dynamics (MD) simulation of the tensile loading process using LAMMPS involves several essential steps. As shown in the Figure 4, firstly, the material’s initial configuration is prepared, typically by constructing a three-dimensional lattice or importing a predefined atomic structure. Next, the system is equilibrated at the desired temperature and pressure using the Nose–Hoover thermostat and barostat, respectively, to achieve a stable starting point. Subsequently, tensile loading is applied to the material by defining appropriate boundary conditions and specifying the displacement rate or strain rate. The simulation then proceeds by integrating the equations of motion using the chosen interatomic potential, such as a classical force field or quantum mechanical model. Throughout the simulation, relevant thermodynamic properties, such as temperature, pressure, and stress, are continuously monitored and recorded to analyze the material’s mechanical response. The simulation is run for a sufficient duration to ensure the material reaches a steady state and exhibits the desired deformation behavior. Finally, the results are meticulously analyzed to obtain key mechanical properties, such as the stress–strain curve. The details of the model are shown in the Table 1. 

### 3.2. Microstructure and Mechanical Properties of Amorphous and Crystal Transition Structure

With increasing heat treatment temperature, amorphous fibers often undergo in situ precipitation of α-Si3N4 crystals. As a reinforcing phase, α-Si3N4 exhibits excellent tensile strength, stiffness, and elastic modulus. When properly distributed between the amorphous and crystalline phases, the in situ formed crystal structures combine with the amorphous matrix, enabling the transfer of strength throughout the ceramic fiber. This reinforcement enhances the overall material strength, enabling it to resist external loads, stress concentration, and crack propagation, thus improving the tensile and flexural strength of the material. The high elastic modulus of the crystal structure, in conjunction with a well-designed transitional interface layer, effectively transmits and disperses stress, as well as absorbs and dissipates energy, thereby imparting exceptional dimensional stability and stability to the ceramic fiber. Adjusting the content of the reinforcing phase and improving the interface bonding between the crystalline and amorphous phases are crucial for enhancing the mechanical performance of ceramic fibers. To investigate this issue, this paper designs three types of amorphous ceramic fiber structures, each with distinct characteristic features. The molecular dynamics method is employed to explore the effects of amorphous structure, crystalline content distribution, and different transitional interface structures on the mechanical properties of the ceramic fiber.

The stability of interfaces is crucial for the reliability and longevity of silicon nitride ceramic fibers. By constructing atomistic-scale transitional interface models [24,25] and employing molecular dynamics, a thorough investigation of the interface’s structure, energy, and dynamic properties can be conducted. This approach enables a deep understanding of the mechanisms governing interface stability, thereby promoting research aimed at enhancing interface stability and improving the material’s resistance to fatigue, corrosion, and oxidation. The stress–strain curve of the amorphous–crystal interface structure during the tensile process can be divided into three stages [26].

**Figure 5:**
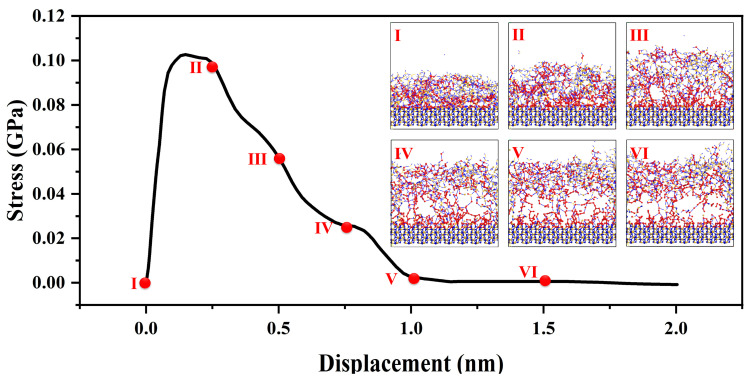
Mechanical properties of amorphous and crystal transition structures; the red ball represents the two atoms where the bond breaks, the figure (I–VI) show the structural transformation process of interface stretching.

As shown in the Figure 5, in the initial stage of tension [27], corresponding to a strain range of 0 to 0.25 nm, the interface exhibits evident elastic behavior, wherein external stress induces relative atomic displacement at the interface while maintaining stable relative positions among atoms. The elastic behavior follows Hooke’s law [20], with stress proportional to strain. At this point, applying tensile strain to the material interface results in a rapid stress response reaching a maximum of 0.1 GPa. As the strain range extends from 0.25 to 1.0 nm, further strain leads to localized plastic deformation at the interface. During this stage, interface atoms experience slip, diffusion, and rearrangement, as the system adapts to increased stress and localized displacement. Such localized plastic deformation causes relative atomic bond displacement and interface movement. When the system can no longer accommodate the passive strain-induced structural changes through interface and bond movement, the strain reaches the fracture strength limit of the interface. Interfacial bonds begin to break, fracture surfaces form, and further promote interface separation. This stage encompasses the entire process of fracture surface formation until the transitional structure, constructed by chemical bonds between amorphous and crystalline structures, is fully disrupted.

For strain ranges greater than 1.0 nanometer, the transitional interface constructed by chemical bonds is entirely disrupted. Further strain does not induce additional stress changes in the system. When strain is applied to the amorphous structure, the system undergoes stress changes to adapt to the structural and energetic changes brought about by the forced strain. During this process, the load generated by the strain is transmitted from the amorphous phase to the crystalline phase. Throughout the entire interface tensile process, failure is concentrated on the side of the amorphous phase. This is attributed to the short-range order present in the amorphous structure, which includes defects and voids, leading to nonuniform density in the macroscopic aggregated structure. As a result, the load transmission process may not uniformly distribute stress, causing stress concentration and facilitating crack formation and propagation during load transmission.

Overall, the investigation reveals the crucial role of interface stability in the mechanical behavior of silicon nitride ceramic fibers. Understanding these mechanisms can significantly contribute to the design and development of more robust and reliable ceramic fiber materials.

### 3.3. Study on Mechanical Properties of Molecular Model of Silicon Nitride Ceramic Fiber

#### 3.3.1. Stress–Strain Curve

**Figure 6:**
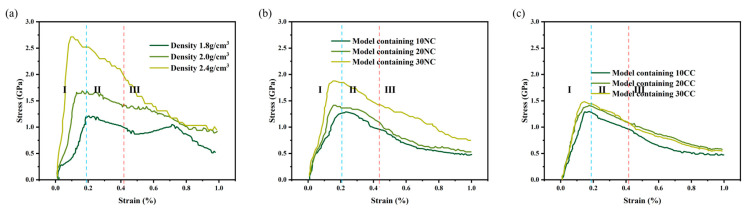
Measurement of stress–strain behavior of silicon nitride ceramic fiber molecular model. (**a**): the tensile curve of different density structure, (**b**): the tensile curve of non-cross-linked structure, (**c**): the tensile curve of cross-linked structure.

The stress–strain curve [28] is commonly used to assess the mechanical properties of materials, such as strength, toughness, and stiffness. It helps determine critical points, optimal working regions, and maximum stress tolerance, providing valuable guidance for material design, selection, and optimization. As shown in the Figure 6, all stress–strain curves exhibit the same characteristic, where they can be reasonably divided into three stages, consistent with experimental characterization curves, indicating the accuracy of this method. By observing the curve characteristics and the microstructural evolution of the molecular model during the tensile process, we identify three stages as follows: elastic region, plastic region, and failure region [29].

1. Elastic region: In the initial stage of tension, the model experiences elastic deformation, and the relative positions of the molecules remain stable. External loading induces relative atomic displacement, but the atomic arrangement remains unchanged, showing linear elastic behavior.
2. Plastic region: As the external stress continues to increase, the model enters the plastic deformation stage. In this stage, relative atomic positions undergo slip, diffusion, and rearrangement. Some stress concentration regions experience Si-N bond breakage to accommodate the increasing load. The changes during this stage are irreversible.
3. Failure region: When the stress in a localized area reaches the model’s fracture strength, stress concentration leads to the formation of fracture surfaces, large-scale rupture of atomic chemical bonds, and the initiation of cracks and fracture surfaces, resulting in material failure.

**Figure 7:**
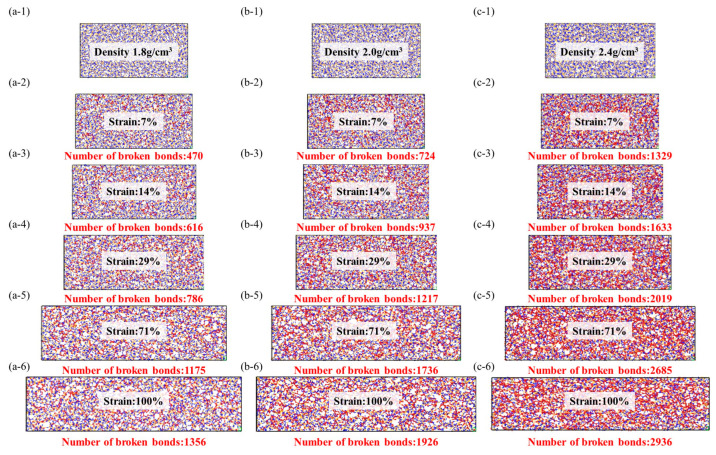
Stress–strain model of amorphous structure, the red ball represents the two atoms where the bond breaks, while the yellow and blue ball represent the crystal structure and there is no bond fracture on it. (**a-1**–**a-6**): Stretching molecular model of density 1.8 g/cm^3^, (**b-1**–**b-6**): Stretching molecular model of density 2.0 g/cm^3^, (**c-1**–**c-6**): Stretching molecular model of density 2.4 g/cm^3^.

**Figure 8:**
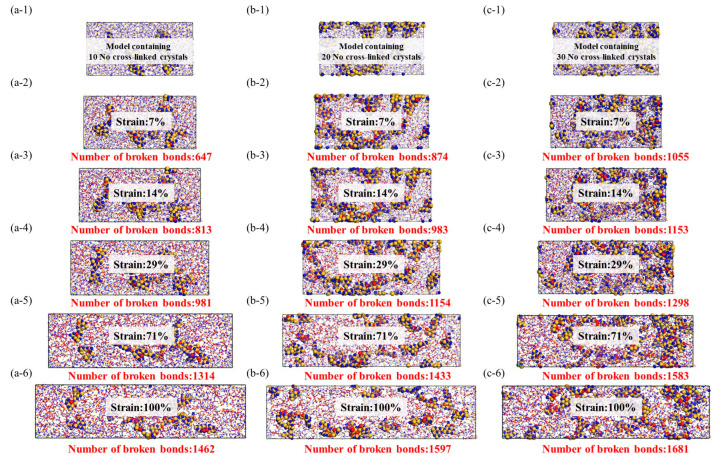
Stress–strain model of noncrosslinked crystal structure, the red ball represents the two atoms where the bond breaks, while the yellow and blue balls represent the crystal structure and there is no bond fracture on it. (**a-1**–**a-6**): Stretching molecular model of 10 no cross-linked crystals, (**b-1**–**b-6**): Stretching molecular model of 20 no cross-linked crystals, (**c-1**–**c-6**): Stretching molecular model of 30 no cross-linked crystals.

**Figure 9:**
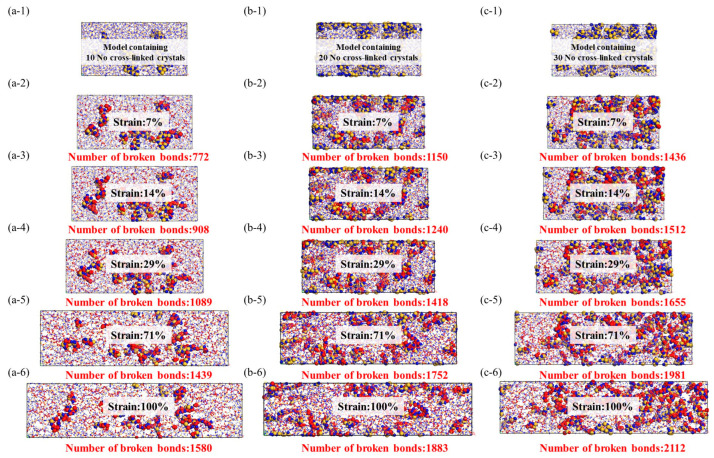
Stress–strain model of crosslinked crystal structure, the red ball represents the two atoms where the bond breaks, while the yellow and blue balls represent the crystal structure and there is no bond fracture on it. (**a-1**–**a-6**): Stretching molecular model of 10 cross-linked crystals, (**b-1**–**b-6**): Stretching molecular model of 20 cross-linked crystals, (**c-1**–**c-6**): Stretching molecular model of 30 cross-linked crystals.

From the data graph Figure 7, Figure 8 and Figure 9, it can be seen that compared to molecular models with densities of 2.0 and 1.8, the addition of a crystalline structure, regardless of the presence of a cross-linked transitional region, effectively enhances the tensile strength of the ceramic structure. The presence of the crosslinked transitional region efficiently transmits and disperses load stress, absorbing this part of the energy and utilizing the higher elastic modulus to organize crack propagation [30]. Therefore, X’s overall performance is stronger than Y’s.

#### 3.3.2. Tensile Fracture Property

Ceramic materials possess unique properties, such as high-temperature resistance, low density, high specific strength, modulus, and hardness, owing to their covalent and ionic bonding structures. However, these advantages are offset by their major weaknesses, including brittleness and poor reliability. The high bond energy contributes to the ceramic structure’s pronounced sensitivity to cracks. In this study, we explore the statistical principles of bond fracture by subjecting the system to controlled gradient deformation within a box. Subsequently, molecular optimization allows for the adjustment of bond length, bond angle, and intermolecular positions, enabling topological optimization of the molecular structure during plastic deformation [31]. This optimization process effectively eliminates part of the stress energy while maintaining a reasonable overall configuration. On the other hand, in stress-concentrated regions, molecules experience constrained movement due to squeezing and deformation, leading to distortion in bond angles and lengths during passive stretching. Since conventional force field strategies do not include bond fracture, we follow the literature’s practices and remove structures with bond lengths exceeding 2.0 during each loading and geometric optimization to mimic bond fracture and crack propagation.

**Figure 10:**
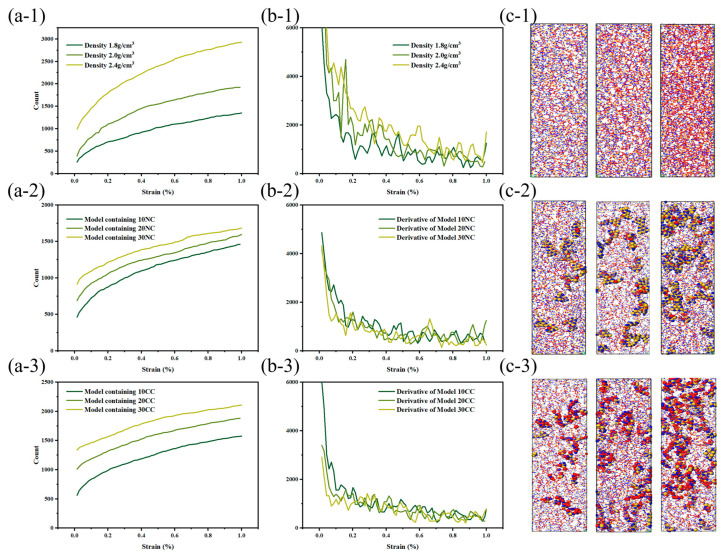
Tensile fracture behavior of silicon nitride ceramic fiber molecular model. (**a-1**–**a-3**): fracture curve of different structures, (**b-1**–**b-3**): first derivative curve of fracture curve, (**c-1**–**c-3**): molecular model of fracture structure.

As shown in the Figure 10, the beginning of bond fracture in stress-concentrated molecular structures indicates the quantity of spline-induced deformation, which is related to material strength. The end of bond fracture signifies the crack propagation capability. During the structural fracture process of necessary load-bearing structures, partial bond fracture occurs in stress-concentrated regions, while the rest of the molecules have more freedom of movement. The increase in free space promotes regulation of bond angles and lengths between molecules [32].

At the start of tensile loading, molecules are immobilized in the material system, and deformation occurs as a necessary condition [33]. At this point, the free volume in the system is zero, preventing molecules from dispersing excess stress through free volume. The beginning of bond fracture is closely related to its strength. In physics and engineering, toughness refers to a material’s ability to absorb energy, undergo extension or deformation, and avoid fracture when subjected to external stress or loads. At the atomic level, toughness is explained by the ability of the material’s molecular structure to absorb stress without fracturing. During the subsequent tensile process, the increase in free volume facilitates a more uniform stress dispersion between molecules. The additional space provided by the free volume allows stress to diffuse and disperse among the molecules, reducing localized stress concentration and decreasing the possibility of crack initiation.

The stress and fracture images clearly illustrate that amorphous structures often exhibit nonuniform and irregular characteristics, lacking long-range order, and containing defects and voids that become stress concentration sites during the application of stress. These stress concentration sites often lead to crack initiation and propagation, resulting in lower tensile strength and higher crack extension in amorphous structures. This tendency increases with higher densities, consistent with the previous literature [34].

In comparison to amorphous structures, the addition of crystalline reinforcement significantly enhances the strength and toughness of fiber structures. Additionally, the non-crosslinked crystalline structure does not generate chemical bonds in the amorphous and crystalline interface regions; instead, it adopts a vacuum structure after the removal of chemical bonds. This provides the amorphous structure with more free space for molecular relaxation during geometric optimization. The non-crosslinked crystalline particles act as rubbery elastic bodies due to their increased surface area. While the inclusion of non-crosslinked crystalline structures does not lead to a significant increase in the system’s strength, the additional free volume introduced by the non-crosslinked interface effectively reduces crack propagation. However, it is worth noting that the introduction of crosslinked crystalline structures enhances the material’s tensile strength, enabling it to achieve higher tensile strength than amorphous structures at lower densities. As illustrated in the figure, the improvement in crosslinked interfaces facilitates more force transmission to the crystalline structure. The colored crystalline regions in red indicate that the interface’s crosslinking enables effective resistance against stress concentration and crack propagation. This results in enhanced tensile strength, flexural rigidity, and fracture toughness of composite materials. In summary, improving the interface effectively inhibits crack propagation, disperses and absorbs stress, and enhances the material’s toughness.

#### 3.3.3. Free Volume and Fracture Behavior

This study explores the influence of free volume on stress distribution within materials. Increased free volume [35] allows stress to disperse more uniformly among molecules, reducing localized stress concentration and lowering the likelihood of crack initiation. Free volume acts as a stress buffer, absorbing and dispersing external stresses [36,37]. It effectively mitigates stress concentration and slows down material failure. Additionally, free volume provides a degree of elastic recovery, allowing materials to return to their original state and reducing deformation in stress-concentrated regions, thereby minimizing permanent deformation and damage.

Based on the structural changes during the tensile process, the variations in free volume can be interpreted as follows:

1. Elastic stage: In the initial stage of tensile loading, the material exhibits elastic deformation, with relatively stable molecular positions and minimal changes in free volume. Although external stress causes molecular displacement and strain, the overall arrangement and gaps between molecules remain largely unchanged.
2. Plastic deformation stage: As external stress increases, the material enters the plastic deformation stage. Here, molecular sliding, diffusion, and rearrangement occur to accommodate the rising stress and localized displacement. These plastic deformation processes may cause relative molecular displacement and reorganization, resulting in changes to the free volume.
3. Fracture stage: Fracture occurs when the material reaches its breaking point. In this stage, the free volume significantly increases as molecular bonds are broken, fracture surfaces form, and previously constrained molecules regain more free space.

**Figure 11:**
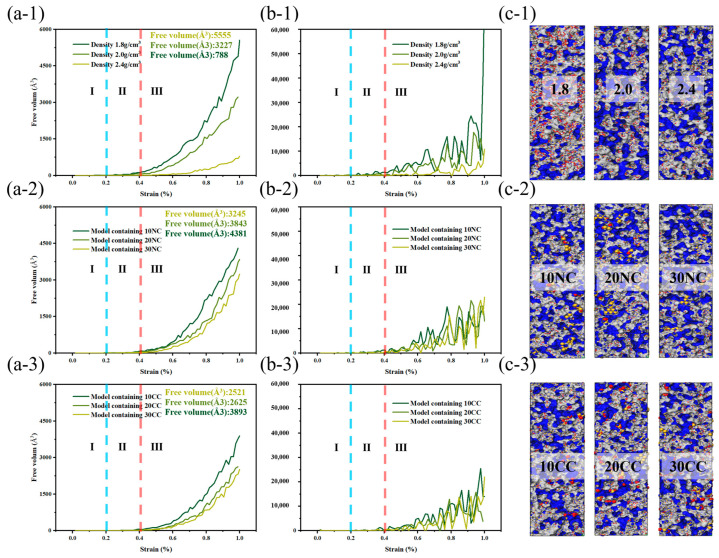
Free volume change of silicon nitride ceramic fiber molecular model during drawing. (**a-1**–**a-3**): free volume curve of different structures, (**b-1**–**b-3**): first derivative curve of free volume curve, (**c-1**–**c-3**): molecular model of free volume.

As depicted in the Figure 11, the incorporation of crystalline structures and interface improvements enable better stress dispersion, energy absorption, and prevention of crack propagation, leading to improved material toughness and fracture toughness. This enhances the ability of composite materials to dissipate and absorb energy when subjected to dynamic and impact loads.

## 4. Conclusions

In this research, a novel approach employing customized molecular dynamics simulations was adopted to construct molecular models of silicon nitride fibers with diverse structures, including amorphous, multi-interface, and crystalline hybrid configurations. The main objective was to explore the relationship between molecular structure composition and the corresponding mechanical properties. The results showcased successful model construction for each fiber type. The mechanical property characterization highlighted essential insights: (1) Amorphous structures exhibited nonuniform and irregular features, leading to stress concentration and increased susceptibility to cracking with higher density. (2) Noninterfacial crosslinked crystalline reinforcement enhanced the material’s free volume and tensile strength, thereby improving its toughness and reducing crack susceptibility. (3) The introduction of crosslinked interfaces effectively improved load transmission efficiency, enhancing tensile strength while reducing crack propagation with increasing density. The enhanced knowledge of silicon nitride fibers’ mechanical properties opens new avenues for material design and optimization, leading to the development of more resilient and high-performance composite materials.

## Figures and Tables

**Table 1:** Model structure composition and unit cell parameters.

Sample	N Content	Si Content	Length *A*(Å)	Length *B*(Å)	Length *C*(Å)
1.8	3200	2400	71.6743	35.33	35.33
2	3200	2400	69.7651	34.3826	34.3826
2.4	3200	2400	65.7881	32.3944	32.3944
10NC	3205	2411	70.6743	35.3371	35.3371
20NC	3260	2444	70.6743	35.3371	35.3371
30NC	3245	2441	70.6743	35.3371	35.3371
10CR	3205	2411	70.2571	35.0131	35.0131
20CR	3260	2444	69.6074	34.8037	34.8037
30CR	3245	2441	69.3168	34.6584	34.6584

## Data Availability

Not applicable.
